# Development of Complement Factor H–Based Immunotherapeutic Molecules in Tobacco Plants Against Multidrug-Resistant *Neisseria gonorrhoeae*

**DOI:** 10.3389/fimmu.2020.583305

**Published:** 2020-10-26

**Authors:** Jutamas Shaughnessy, Y Tran, Bo Zheng, Rosane B. DeOliveira, Sunita Gulati, Wen-Chao Song, James M. Maclean, Keith L. Wycoff, Sanjay Ram

**Affiliations:** ^1^Division of Infectious Diseases and Immunology, University of Massachusetts Medical School, Worcester, MA, United States; ^2^Planet Biotechnology, Inc., Hayward, CA, United States; ^3^Department of Systems Pharmacology and Translational Therapeutics, Perelman School of Medicine, University of Pennsylvania School of Medicine, Philadelphia, PA, United States

**Keywords:** *Neisseria gonorrhoeae*, gonorrhea, factor H, immunotherapeutic, Fc fusion protein, *Nicotiana benthamiana*, complement, factor H (FH)

## Abstract

Novel therapeutics against the global threat of multidrug-resistant *Neisseria gonorrhoeae* are urgently needed. Gonococci possess several mechanisms to evade killing by human complement, including binding of factor H (FH), a key inhibitor of the alternative pathway. FH comprises 20 short consensus repeat (SCR) domains organized in a head-to-tail manner as a single chain. *N. gonorrhoeae* binds two regions in FH; domains 6 and 7 and domains 18 through 20. We designed a novel anti-infective immunotherapeutic molecule that fuses domains 18–20 of FH containing a D-to-G mutation in domain 19 at position 1119 (called FH*) with human IgG1 Fc. FH*/Fc retained binding to gonococci but did not lyse human erythrocytes. Expression of FH*/Fc in tobacco plants was undertaken as an alternative, economical production platform. FH*/Fc was expressed in high yields in tobacco plants (300–600 mg/kg biomass). The activities of plant- and CHO-cell produced FH*/Fc against gonococci were similar *in vitro* and in the mouse vaginal colonization model of gonorrhea. The addition of flexible linkers [e.g., (GGGGS)_2_ or (GGGGS)_3_] between FH* and Fc improved the bactericidal efficacy of FH*/Fc 2.7-fold. The linkers also improved PMN-mediated opsonophagocytosis about 11-fold. FH*/Fc with linker also effectively reduced the duration and burden of colonization of two gonococcal strains tested in mice. FH*/Fc lost efficacy: i) in *C6^−/−^* mice (no terminal complement) and ii) when Fc was mutated to abrogate complement activation, suggesting that an intact complement was necessary for FH*/Fc function *in vivo*. In summary, plant-produced FH*/Fc represent promising prophylactic or adjunctive immunotherapeutics against multidrug-resistant gonococci.

## Introduction

Gonorrhea is caused by the Gram-negative bacterium *Neisseria gonorrhoeae*. Each year about 87 million new cases of gonorrhea occur worldwide (1). Gonorrhea commonly manifests as cervicitis, urethritis, proctitis, and conjunctivitis and can result in serious sequelae in woman including infertility, ectopic pregnancy, and chronic pelvic pain. Concomitant infection with HIV and gonorrhea enhances the rate of HIV transmission (2–4). Over the years *N. gonorrhoeae* has become resistant to almost every antibiotic that has been used for treatment (5, 6). The recent emergence of azithromycin-resistant isolates in several countries (7–10) could render the first-line therapy, ceftriaxone plus azithromycin, recommended by the Centers for Disease Control and Prevention (https://www.cdc.gov/std/tg2015/default.htm), ineffective in the near future.

In light of rapidly emerging multidrug-resistant *N. gonorrhoeae* worldwide, development of safe and effective vaccines and novel therapeutics against gonorrhea is a high priority (11). An approach for developing new and effective therapeutics against gonorrhea is to target key bacterial virulence mechanisms. One of these is the ability of *N. gonorrhoeae* to bind factor H (FH), a key inhibitor of the alternative pathway of complement (12). FH comprises 20 short consensus repeat (SCR) domains that are organized as a single chain (13). *N. gonorrhoeae* binds FH through domains 6 and 7 (14, 15) and the C-terminal domains 18 through 20 (12, 16). We previously designed a novel anti-infective immunotherapeutic molecule combining the *N. gonorrhoeae*-binding C-terminal domains 18–20 of FH, with a D to G mutation at position 1119 in FH (termed FH*) to minimize binding to human tissue while retaining binding to *N. gonorrhoeae*, with human IgG1 Fc (the antibody-like effector region of the modified molecule [termed FH*/Fc]) (17). We showed that FH*/Fc possessed complement-dependent bactericidal activity against gonococci *in vitro* and shortened the duration and diminished bacterial loads in the mouse model of vaginal colonization (17).

One of the important variables that we considered when we designed FH*/Fc is the choice of linker length and sequence (18–20). Linkers may offer some advantages for the production of fusion protein, such as improving biological activity and increasing expression yield (19). One of the most commonly used flexible linkers has the sequence of (Gly-Gly-Gly-Gly-Ser)n, where “n” can be optimized to achieve appropriate separation of the functional domains (18). We previously used a simple AAAGG-containing linker between FH* and Fc domain (17). In this work, we explored the role of different linker lengths in the efficacy of protein by generating FH*/Fc with no linker, AAAGG, (GGGGS)_2_, and (GGGGS)_3_. In addition, we expressed these molecules in tobacco plants because of the ability for large scale production, low cost and the absence of animal products (21–24). We also compared the functions of these molecules to CHO-cell-produced FH*/Fc.

## Materials and Methods

### Bacterial Strains

Strains F62 (25), Ctx-r(Spain) (similar to strain F89) (26), H041 (also known as World Health Organization reference strain X) (27, 28), MS11 (29), UMNJ60_06UM (NJ-60) (30), and FA1090 (31) have all been described previously. Strains Ctx-r(Spain), H041, and NJ-60 are resistant to ceftriaxone. Opacity protein (Opa)–negative mutants of FA1090 (32) (all *opa* genes deleted) have been described previously.

### Expression and Purification of FH/Fc Fusion Proteins in Tobacco Plants

A nucleotide sequence encoding human FH SCR18-20 (GenBank accession no. NP_000177) [aa 1048-1231, incorporating the D1119G mutation (33)], designed to employ optimal codon usage for expression in *Nicotiana benthamiana*, was synthesized by GENEWIZ (South Plainfield, NJ). This sequence (and the encoded protein fragment) was designated FH*.

The synthetic FH* sequence was cloned into the plant binary expression vector pTRAkc (34) upstream and in-frame with codon-optimized hinge, C_H_2 and C_H_3 domains from human IgG1 (hFc) and downstream of the signal peptide of the murine mAb24 heavy-chain (lph) (35). Additional clones encoding N-terminal amino acid extensions to the FH* sequence or linkers between FH* and Fc were made using overlap extension PCR. The molecular constructs that were assembled are listed in **Table 1**. Throughout the text these are referred to by *Agrobacterium tumefaciens* strain number.

**Table 1:** Description of plant-produced FH*/Fc molecules.

Strain	Modifications	Binary expression vector name
S2366	AAAGG linker	pTRAk-c-lph-FH*-(AAAGG)-hFc
S2368	(GGGGS)_2_ [(G_4_S)_2_] linker	pTRAk-c-lph-FH*-(GGGGS)2-hFc
S2370	(GGGGS)_3_ [(G_4_S)_3_] linker	pTRAk-c-lph-FH*-(GGGGS)3-hFc
S2381	no linker	pTRAk-c-lph-FH*-hFc
S2477	N-terminal TS	pTRAk-c-lph-(TS)FH*-(G_4_S)_2_-hFc
S2493	N-terminal TS “complement-inactive”	pTRAk-c-lph-(TS)FH*-(G_4_S)_2_-hFc(D270A/K322A)

Transient expression of recombinant proteins was accomplished by whole-plant vacuum infiltration (36) of *N. benthamiana* ΔXT/FT (37) using *A. tumefaciens* GV3101 (pMP90RK) (38) containing one of the binary expression vectors, co-infiltrated with *A. tumefaciens* GV3101 (pMP90RK) containing the binary vector pTRAkc-P19, encoding the post-transcriptional silencing suppressor P19 (39). Glycoproteins produced in *N. benthamiana* ΔXT/FT contain almost homogeneous N-glycan species without plant-specific β1,2-xylose and α1,3-fucose residues (37). After infiltration, the plants were maintained in a grow room under continuous light at 25°C for 5–7 days prior to harvest and protein purification.

Leaves were collected 5–7 days after vacuum infiltration and frozen at −80°C until use. Purification of FH*/Fc fusion proteins was accomplished using a protocol previously used with another plant-produced Fc fusion (40), which incorporates affinity chromatography with Protein A-MabSelect SuRe (GE HealthCare). Purified proteins were concentrated to ≥2 mg/ml using 10 kDa cut-off centrifugal concentrators, buffer exchanged into PBS, and rendered sterile by filtration through 0.22-μm PES membrane filters. Protein concentrations were quantified using absorption at 280 nm and extinction coefficients predicted from the amino acid sequences.

Purified protein samples were analyzed using standard methods. Samples were subjected to SDS-polyacrylamide gel electrophoresis (under reducing and non-reducing conditions) on 4%–20% Mini-PROTEAN^®^ TGX Stain-Free™ Protein Gels (Bio-Rad, Hercules, CA). Gel images were obtained using a Bio-Rad Gel Doc EZ imaging system.

### Expression and Purification of FH/Fc Fusion Proteins in CHO Cells

Cloning, expression in CHO cells and purification from cell culture supernatants of a chimeric protein comprising human FH (HuFH) domains 18–20 (D1119G) fused to the hinge, C_H_2 and C_H_3 domains of human IgG1 (hFc) has been described previously (17). Protein concentrations were determined using absorption at 280 nm and the BCA protein Assay kit (Pierce); mass was determined by Coomassie Blue staining of proteins separated by SDS-PAGE.

### Human Complement

IgG- and IgM-depleted normal human serum (human complement) was purchased from Pel-Freez.

### Antibodies

Anti-human IgG–FITC was from Sigma-Aldrich and was used at a dilution of 1:100 in HBSS containing 0.15 mM CaCl_2_ and 1 mM MgCl_2_ (HBSS^++^) and 1% BSA (HBSS^++^/BSA) in flow cytometry assays. Goat anti-human FH, alkaline phosphatase conjugated anti-human IgG (Southern Biotechnology), and donkey anti-goat IgG were used in Western blots a dilution of 1:1,000 in PBS with 5% non-fat dry milk.

### Flow Cytometry

Binding of FH*/Fc to bacteria was measured by flow cytometry as described previously (17). Data were acquired on a BD LSR II flow cytometer, and data were analyzed using FlowJo software.

### Serum Bactericidal Assay

Serum bactericidal assays using bacteria grown in gonococcal liquid media supplemented with CMP-Neu5Ac (2 µg/ml) were performed as described previously (17, 41). Approximately, 2,000 colony forming units (CFUs) of *N. gonorrhoeae* were incubated with 20% human complement [IgG and IgM depleted normal human serum (Pel-Freez)] in the presence or the absence of the FH*/Fc fusion protein (concentration indicated for each experiment). The final volume of the bactericidal reaction mixture was 150 µl. Aliquots of 25µl reaction mixtures were plated onto chocolate agar in duplicate at the beginning of the assay (t_0_) and again after incubation at 37°C for 30 min (t_30_). Survival was calculated as the number of viable colonies at t_30_ relative to t_0_.

### Opsonophagocytosis Assay

Opsonophagocytic killing of gonococci with freshly isolated human polymorphonuclear leukocytes (PMNs) was performed as described previously (15, 17). Briefly, heparinized venous blood was obtained from a healthy adult volunteer in accordance with a protocol approved by the Institutional Review Board. PMNs were isolated using Mono-Poly Resolving Medium (MP Biomedicals) according to the manufacturer’s instructions. Isolated PMNs were washed and suspended in HBSS without added divalent cations, counted, and diluted to 1 × 10^7^/ml in HEPES-buffered RPMI 1640 medium supplemented with l-glutamine and 1% heat-inactivated FBS. To measure survival of gonococci in the presence of PMNs, Opa-negative mutant of *N. gonorrhoeae* strain FA1090 was added to 1 × 10^6^ PMNs at a multiplicity of infection of 1 (two bacteria to one PMN). Opa-negative (Opa^−^) *N. gonorrhoeae* was used because select Opa proteins serve as ligands for human carcinoembryonic Ag–related cell adhesion molecule 3 (CEACAM3) that is expressed by PMNs and results in phagocytosis (42). FH*/Fc was added at different concentrations, followed by 10% human complement (Pel-Freez). The reaction mixtures were incubated for 60 min at 37°C in a shaking water bath. Bacteria were serially diluted and plated at 0 and 60 min on chocolate agar plates. Percentage survival of gonococci in each reaction was calculated as a ratio of CFU at 60 min to CFU at the start of the assay (0 min).

### Mouse Strains

Human FH and C4b-binding protein (C4BP) (FH/C4BP) transgenic mice) in a BALB/c background have been described previously (43). FH/C4BP Tg mice express levels of FH and C4BP that are comparable to those found in human serum and show similar responses to a variety of stimuli as wild-type (wt) BALB/c mice (43). Wild-type C57BL/6 mice were purchased from Jackson laboratories. Construction and characterization of *C6^−/−^* mice (C57BL/6 background) have been described previously (44).

### Mouse Vaginal Colonization Model of Gonorrhea

Use of animals in this study was performed in strict accordance with the recommendations in the *Guide for the Care and Use of Laboratory Animals* by the National Institutes of Health. The protocol was approved by the Institutional Animal Care and Use Committee at the University of Massachusetts Medical School. Female mice 6–8 weeks of age in the diestrus phase of the estrous cycle were started on treatment with 0.1-mg Premarin (Pfizer; conjugated estrogens) in 200 μl of water given s.c. on each of 3 days: −2, 0, and +2 (2 days before, the day of, and 2 days after inoculation) to prolong the estrus phase of the reproductive cycle and promote susceptibility to *N. gonorrhoeae* infection. Antibiotics (vancomycin and streptomycin) ineffective against *N. gonorrhoeae* were also used to reduce competitive microflora (45). Mice were infected on day 0 with either strain H041 or FA1090 (inoculum specified for each experiment). Mice were treated daily with 1 or 10 μg FH*/Fc intravaginally from day 0 until the conclusion of the experiment or were given a corresponding volume of PBS (vehicle controls).

### Statistical Analysis

Concentration-dependent complement-mediated killing by FH/Fc across strains was compared using two-way ANOVA. Experiments that compared clearance of *N. gonorrhoeae* in independent groups of mice estimated and tested three characteristics of the data (15, 17, 46): time to clearance, longitudinal trends in mean log_10_ CFU, and the cumulative CFU as area under the curve (AUC). Statistical analyses were performed using mice that initially yielded bacterial colonies on days 1 and/or 2. Median time to clearance was estimated using Kaplan-Meier survival curves; times to clearance were compared between groups using the Mantel-Cox log-rank test. Mean log_10_ CFU trends over time were compared between groups using two-way ANOVA and Dunnett’s multiple comparison test. The mean AUC (log_10_ CFU versus time) was computed for each mouse to estimate the bacterial burden over time (cumulative infection). The means under the curves of two groups were compared using the nonparametric Mann-Whitney test because distributions were skewed or kurtotic. The Kruskal-Wallis equality-of-populations rank test was also applied to compare more than two groups in an experiment.

## Results

### Production of FH*/Fc Molecules in *Nicotiana benthamiana*

**Figure 1:**
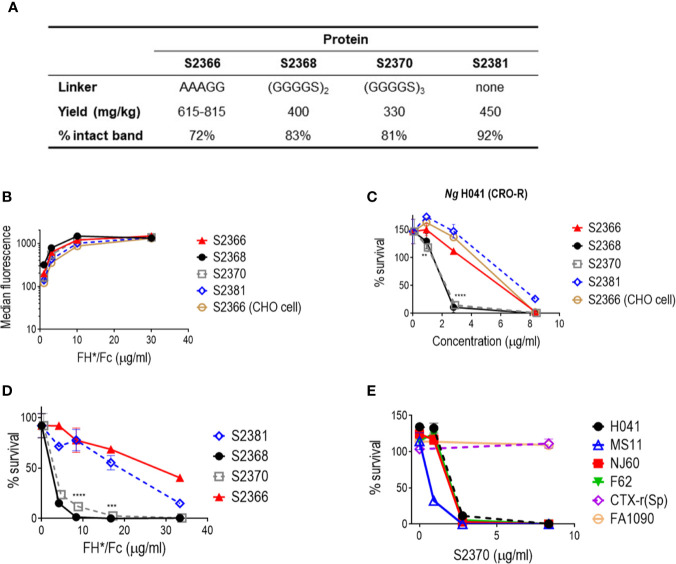
Effect of linkers in efficacy of FH/Fc produced in *N. benthamiana* against *N. gonorrhoeae in vitro*. **(A)** Yields and stability of the four human IgG1 Fc variants produced in tobacco plants. **(B)** Binding of FH*/Fc fusion proteins to sialylated *N. gonorrhoeae* H041. CHO cell-produced FH*/Fc that was used in previous studies was used as a comparator. **(C)** Bactericidal activity of the FH*/Fc fusion proteins against *N. gonorrhoeae* H041. S2368 [(G_4_S)_2_ linker] and S2370 [(G_4_S)_3_ linker] show improved activity. **(D)** Comparison of the opsonophagocytic activity of S2368, S2370 and S2381 (no linker) against *N. gonorrhoeae* FA1090. Presence of the G_4_S linker improves function. **(E)** Activity of S2370 against six sialylated strains of *N. gonorrhoeae*.

We cloned a plant codon-optimized FH* DNA sequence upstream and in-frame with sequences encoding the hinge, CH_2_ and CH_3_ domains (Fc) of human IgG1 in a plant expression vector, then produced the FH*/Fc using a rapid *N. benthamiana* expression system. One variant (S2366) included an AAAGG linker between FH* and Fc, resulting in the same protein that had previously been expressed in CHO cells (17). We also produced three new FH*/hFc variants containing either no linker (S2381) or two or three copies of a GGGGS (G_4_S) linker (S2368 and S2370, respectively). Yield of these proteins following Protein A affinity chromatography ranged from 300 to 600 mg per kg plant fresh weight (**Figure 1A**). Characterization of the plant produced proteins by protein staining of SDS-PAGE gels and western blotting with anti-human FH is shown in **Supplemental Figure S1**.

### Effect of Linkers on Efficacy of FH*/Fc

We initially characterized four FH*/Fc molecules made in tobacco plants: FH*/Fc without a linker, or with AAAGG, two G_4_S or three G_4_S linkers (called (G_4_S)_2_ and (G_4_S)_3_, respectively). FH*/Fc with AAAGG linker made in CHO cells was used as a control. As we expected, since all proteins possessed the same FH* sequence they showed similar binding to *N. gonorrhoeae* strain H041 when tested at dilutions ranging from 1.1 to 30 µg/ml (**Figure 1B**). In human complement-dependent bactericidal assays using *N. gonorrhoeae* strain H041, S2368 and S2370 (FH*/Fc with (G_4_S)_2_ and (G_4_S)_3_, respectively) showed improved bactericidal activities compared to S2366 (FH*/Fc with AAAGG) or S2381 (FH*/Fc without a linker) (**Figure 1C**). The concentrations required for 50% bactericidal activity (BC_50_) were lower for S2368 and S2370 than for S2366 and S2381 (BC_50_ of 2.1 µg/ml with S2368 and S2370 vs. 5.9 and 7.2 µg/ml with S2366 and S2381, respectively). FH*/Fc with AAAGG generated in CHO cells or tobacco plants (S2366) showed similar bactericidal activity (BC_50_ of 6.3 and 5.9 µg/ml, respectively). S2381 (no linker) showed the least killing.

We next evaluated the effect of linkers on opsonophagocytic activity. We have shown previously that FH*/Fc made in CHO cells enhanced complement-dependent killing by PMN (17). In this experiment, we used an Opacity protein negative (Opa-) mutant derivative of *N. gonorrhoeae* strain FA1090, where all 11 *opa* genes have been inactivated, to eliminate Opa-CAECAM3 induced uptake of gonococci by PMNs (42). As shown in **Figure 1D**, S2368 and S2370 enhanced PMN-mediated killing significantly more than S2366 or S2381 (BC_50_ of 2.3 and 2.6 µg/ml with S2368 and S2370 vs. 27.4 and 19.1 µg/ml with S2366 and S2381, respectively).

Collectively, the data above showed that S2368 and S2370 [(G_4_S)_2_ and (G_4_S)_3_ linkers, respectively] improved bactericidal and PMN-mediated opsonophagocytic killing about 2.7- and 11- fold, respectively, compared to S2366. We chose S2370 for further bactericidal testing using five additional gonococcal strains (**Figure 1E**) and observed killing of four of the six strains tested [H041, NJ60, F62, and MS11, but not FA1090 or CTX-r(Sp)]. These six strains showed the same pattern of susceptibility to FH*/Fc with the AAAGG linker produced in CHO cells (17).

### Efficacy of S2370 Against *N. gonorrhoeae* in the Mouse Vaginal Colonization Model

We next evaluated the efficacy of S2370 against *N. gonorrhoeae* in the mouse vaginal colonization model of gonorrhea using FH/C4BP transgenic mice. We used two strains that differed in their susceptibility to killing in the human complement-dependent bactericidal assay; sensitive strain H041 and resistant strain FA1090 (**Figure 1E**).

**Figure 2:**
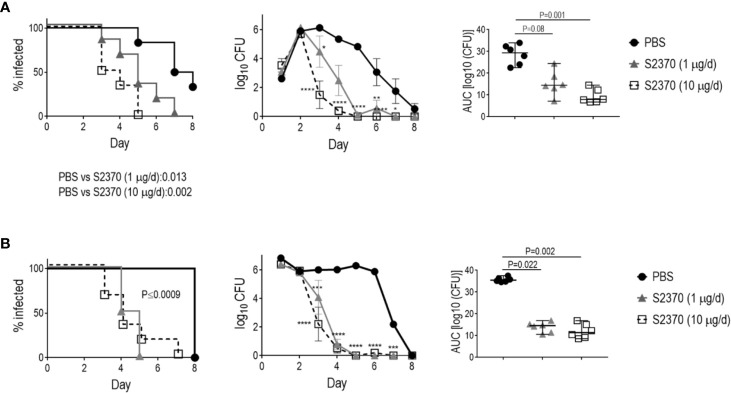
Efficacy of S2370 against *N. gonorrhoeae* H041 in human FH/C4BP transgenic mice. Premarin^®^-treated 6- to 8-week-old human FH/C4BP transgenic mice (n = 6/group) were infected with either 10^6^ CFU **(A)** or 10^7^ CFU **(B)** *N. gonorrhoeae* strain H041. Mice were treated daily (starting 2 h before infection) intravaginally either with PBS (vehicle control) or with 1 µg or 10 µg of FH*/Fc molecule S2370. *Left graphs*: Kaplan Meier curves showing time to clearance, analyzed by the Mantel-Cox (log-rank) test. Significance was set at 0.017 (Bonferroni’s correction for comparisons across three groups). *Middle graphs*: log_10_ CFU versus time. X-axis, day; Y-axis, log_10_ CFU. Comparisons of the CFU over time between each treatment group and the respective saline control was made by two-way ANOVA and Dunnett’s multiple comparison test. *P < 0.05; **P < 0.01; ***P < 0.001; ****P < 0.0001. *Right graphs*: bacterial burdens consolidated over time (area under the curve [log_10_ CFU] analysis). The three groups were compared by one-way ANOVA using the non-parametric Kruskal-Wallis equality of populations rank test. The χ^2^ with ties were 12.12 (P = 0.0002) and 11.94 (P = 0.0002) for the graphs in panels **(A, B)**, respectively. Pairwise AUC comparisons across groups was made with Dunn’s multiple comparison test.

**Figure 3:**
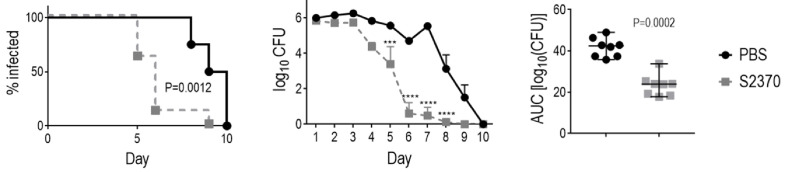
Efficacy of S2370 (FH/Fc with (GGGGS)_3_ linker) against *N. gonorrhoeae* FA1090 in human FH/C4BP transgenic mice. Premarin^®^-treated 6 week-old human FH/C4BP transgenic mice (n = 8/group) were infected with 4 × 10^7^ CFU *N. gonorrhoeae* strain FA1090. Mice were treated daily (starting 2 h before infection) intravaginally either with PBS (vehicle control) or with 10 µg of FH*/Fc molecule S2370. Left graph: Kaplan Meier curves showing time to clearance, analyzed by the Mantel-Cox (log-rank) test. Middle graph: log_10_ CFU versus time. X-axis, day; Y-axis, log_10_ CFU. Comparisons of the CFU over time between each treatment group and the respective saline control was made by two-way ANOVA and Dunnett’s multiple comparison test. ***P < 0.001; ****P < 0.0001. Right graphs: bacterial burdens consolidated over time (area under the curve [log_10_ CFU] analysis). Comparisons were made by Mann-Whitney’s non-parametric test.

As shown in **Figure 2**, S2370 given daily intravaginally at doses of either 1 or 10 µg/d significantly attenuated both the duration and the burden of gonococcal vaginal colonization compared to vehicle control treated groups, when challenged with either 10^6^ (**Figure 2A**) or 10^7^ CFU (**Figure 2B**) of strain H041. Overall, there were no significant differences in clearance between the 1 or 10 µg doses. S2370 was also efficacious against strain FA1090 in FH/C4BP transgenic mice when administered intravaginally at a dose of 10 µg/d (**Figure 3**).

### Capping the N-terminal Cys in FH*/Fc Improves Protein Yields and Retains Function

**Figure 4:**
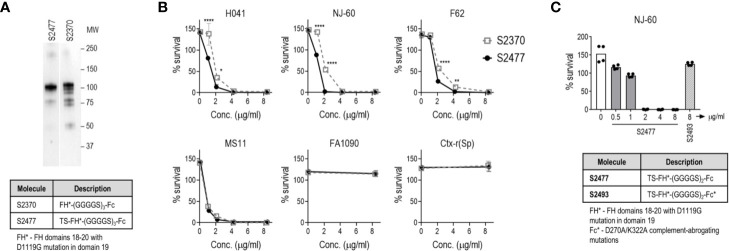
Improved stability and efficacy of FH*/Fc bearing two amino acids (TS) at the N-terminus (S2477) *in vitro*. **(A)** S2477 shows fewer degradation products compared to S2370. Western blot of purified S2477 (lane 1) and S2370 (lane 2) using anti-human IgG alkaline phosphatase as the detection reagent. Note that irrelevant lanes between lanes 1 and 2 have been excluded. MW, molecular weight (kDa). **(B)** S2477 (TS-FH*-(G_4_S)_2_/Fc) and S2370 (FH*-(G_4_S)_3_/Fc) (concentrations indicated on the X-axis) were incubated with sialylated strains H041, NJ-60, F62, MS11, FA1090, and Ctx-r(Sp) and complement and survival at 30 min (relative to 0 min) was measured in a bactericidal assay. Comparisons were made by two-way ANOVA. *P < 0.05; **P < 0.01; ****P < 0.0001. **(C)** Complement-dependent bactericidal efficacy of S2477 against *N. gonorrhoeae* strain NJ-60. Negative controls included bacteria incubated with complement alone (open bar on left) and bacteria incubated with 8 µg/ml S2493 (TS-FH*-(G_4_S)_2_/Fc-D270A/K322A (complement-inactive Fc mutations); hatched bar on right).

We observed that concentration and sterile filtration of all variants of FH*/Fc resulted in dramatic losses of protein; close to 50% versus the ~20% loss seen with other plant-produced Fc fusions (40, 47). A distinctive feature of FH*/Fc is the presence of an N-terminal cysteine. Proteins with N-terminal cysteines are able to undergo a reaction called native chemical ligation, whereby the cysteine reacts with free thioester groups (48, 49). We suspected this might be responsible for the protein loss during concentration. We therefore designed, expressed, and purified a new FH*/Fc (S2477) with two additional amino acids (TS) that are normally N-terminal to the cysteine in the native FH sequence, which overcame the previously noted loss during purification. As shown in **Figure 4A**, S2477 showed fewer degradation products after purification compared to S2370.

A comparison of the bactericidal activity of S2370 and S2477 against six strains of *N. gonorrhoeae* [H041, NJ-60, F62, MS11, FA1090, and Ctx-r(Sp)] grown in media containing CMP-Neu5Ac to sialylate LOS showed that S2477 has slightly better activity than S2370 (**Figure 4B**). The efficacy of S2477 against another ceftriaxone-resistant isolate, NJ60, was also confirmed (BC_50_ of 1.5 µg/ml) (**Figure 4C**). By comparison, S2493 [a derivative of S2477 that contained D270A and K322A in Fc, abrogating C1q binding (50)] was included as a negative control and showed no killing (**Figure 4C**).

### S2477 Requires an Intact Terminal Complement Pathway for Efficacy

**Figure 5:**
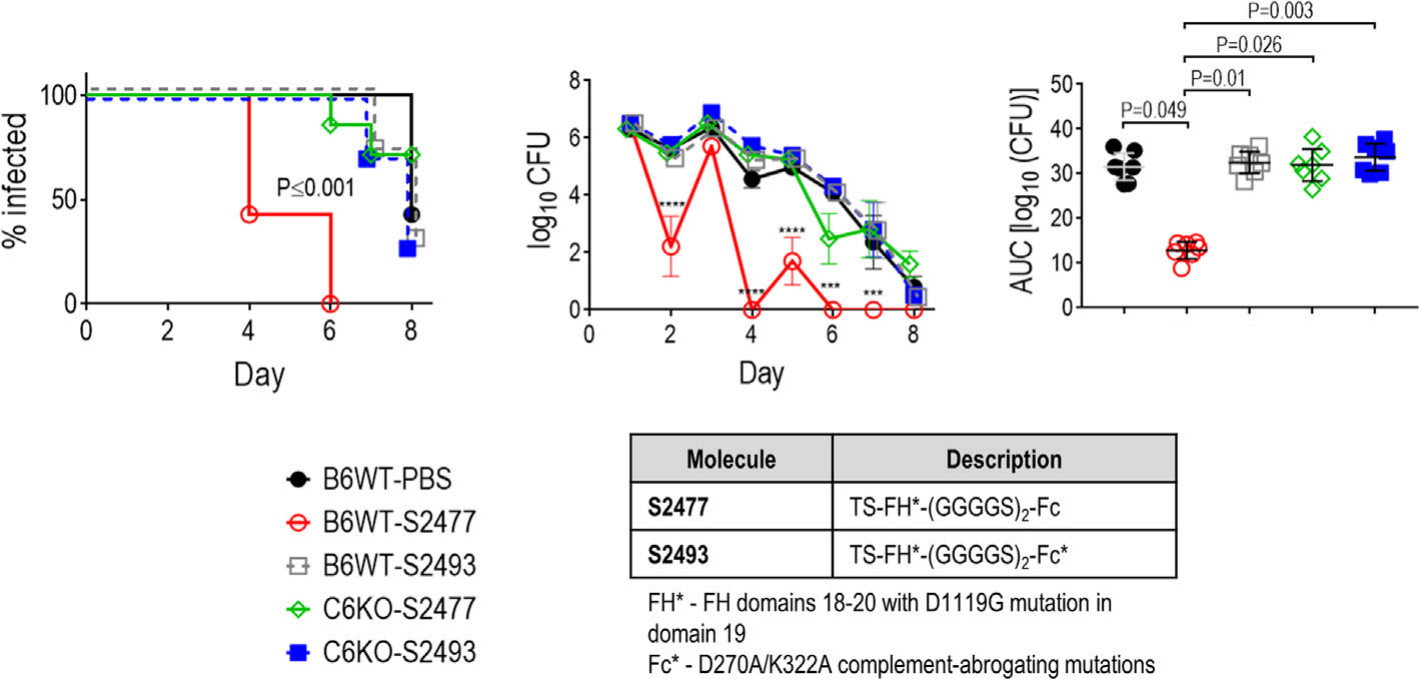
Terminal complement is required for efficacy of FH/Fc against *N. gonorrhoeae* H041 *in vivo*. The activities of S2477 (TS-FH*/Fc with (G_4_S)_2_ linker) and S2493 (the corresponding FH/Fc molecule with D270A/K322A mutations in Fc that abrogates complement activation) were tested in *C6^−/−^* mice or wt C57BL/6 control mice. Mice (n = 7/group) were infected with 4.2 × 10^6^ CFU *N. gonorrhoeae* H041 and treated daily (starting 2 h before infection) with 5 µg of the indicated FH/Fc protein intravaginally; control animals received PBS. *Left graph*: Kaplan Meier curves showing time to clearance, analyzed by the Mantel-Cox (log-rank) test. Significance was set at 0.005 (Bonferroni’s correction for comparisons across five groups). *Middle graph*: log_10_ CFU versus time. X-axis, day; Y-axis, log_10_ CFU. Comparisons of the CFU over time between each treatment group and the respective PBS control was made by two-way ANOVA and Dunnett’s multiple comparison test. ***P < 0.001; ****P < 0.0001. *Right graph*: bacterial burdens consolidated over time (area under the curve [log_10_ CFU] analysis). The five groups were compared by one-way ANOVA using the non-parametric Kruskal-Wallis equality of populations rank test. The χ^2^ with ties was 17.15 (P = 0.0018). Pairwise AUC comparisons across groups was made with Dunn’s multiple comparison test.

C1q engagement by Fc is critical for the activity of CHO cell-produced FH*/Fc (15), suggesting that the classical complement pathway is required for efficacy of FH*/Fc. To determine whether complement alone acting through killing by membrane attack complex (MAC) insertion was necessary and sufficient for efficacy of FH*/Fc, we used *C6^−/−^* mice (44). C6 is the second step in the formation of the C5b-9 MAC pore. While *C6^−/−^* mice lack the capacity to form MAC pores, they can generate C5a, which is important for chemotaxis of PMNs and opsonophagocytic killing of Neisseriae (51, 52). Wild-type C57BL/6 control mice or *C6^−/−^* mice (n = 6/group) were infected with H041 and treated with either S2477 or S2493 (each given at 5µg intravaginally daily, starting on day 0, through day 7) or PBS vehicle control (**Figure 5**). Although S2477 was efficacious in WT C57BL/6 mice, all efficacy was lost in *C6^−/−^* mice. FH*/Fc that lacked the ability to activate complement (S2493) was inactive in both *C6^−/−^* and wt mice. Taken together, these data show that complement alone is necessary and sufficient for efficacy of FH*/Fc in the mouse vaginal colonization model of gonorrhea.

## Discussion

*N. gonorrhoeae* has developed resistance to almost every antibiotic used for treatment and poses an urgent threat to human health worldwide. The “Global action plan to control the spread and impact of antimicrobial resistance in *N. gonorrhoeae*” emphasizes the need for novel approaches to prevent and treat gonorrhea (53). The complement system is a critical component of innate immune defense that is central to controlling bacterial infections. *N. gonorrhoeae* have evolved several strategies to escape complement, including binding of FH, a key inhibitor of the alternative pathway of complement (12, 54). Sialylation of gonococcal LOS occurs in humans (55) and also during experimental infection of mice (56). Loss of the ability to sialylate its LOS is associated with a significant decrease in the ability of gonococci to colonize mice (56, 57). Targeting a gonococcal virulence factor has a distinct advantage over conventional antibiotics because resistance, if it were to develop, would result in a less fit organism due to loss of the virulence factor.

Gonococcal surface antigens show extensive antigenic and phase variability (58, 59). Thus, the identification of protective epitopes that are shared by a wide array of strains has been challenging. To overcome this obstacle, we designed an immunotherapeutic molecule combining the gonococcal-binding C-terminal domains 18, 19, and 20 of FH with human IgG1 Fc. This molecule has the advantage of targeting a broad array of gonococcal isolates. Introducing a D-to-G mutation at position 1119 in FH domain 19 (FH*) abrogated lysis of human RBCs that was seen when unmodified FH domains 18–20 were fused to Fc, while retaining binding to and activity against gonococci *in vitro* and *in vivo* (17).

In this study, we examined the efficacy of tobacco plant-produced FH*/Fc. Tobacco plants have been used for over three decades to produce antibodies and proteins (60). The tobacco plant expression system has advantages over mammalian cells because of the scalability of production, the potentially low costs and the absence of animal viruses or prions (22). FH*/Fc molecules were expressed in high yields in tobacco plants (>300 mg/kg biomass). Plant-produced FH*/Fc showed activity against *N. gonorrhoeae* that was comparable with CHO cell-produced FH*/Fc.

Linkers between the binding domain and Fc can positively impact production and/or function of fusion proteins (19, 20, 61). Accordingly, insertion of (G_4_S)_2_ and (G_4_S)_3_ flexible linkers between FH* and Fc improved the functional efficacy of FH*/Fc, evidenced by approximately 3- and 11-fold increases in bactericidal activity and PMN-mediated killing, respectively, compared to FH*/Fc with an AAAGG linker. The (G_4_S)_3_ linker-containing FH*/Fc was efficacious in mice against ceftriaxone-resistant isolate H041 when given topically at a dose as low as 1 µg/d.

Complement is a central arm of innate immune defenses against Neisserial infections. Defects of terminal complement components (C5 through C9) are associated with increased risk from invasive Neisserial infections, including disseminated gonococcal infection (62–68). We used mice deficient in complement C6 (*C6^−/−^* mice) to assess the role of terminal pathway in enabling FH*/Fc to clear *N*. *gonorrhoeae*. The opsonophagocytic activity in *C6^−/−^* mice is intact because they can generate C5a, a chemotaxin shown to be important for killing of *N. meningitidis* in blood where C7 function was blocked (52). FH*/Fc lost activity in *C6^−/−^* mice, suggesting terminal complement was required for FH*/Fc activity. The lack of FH*/Fc activity in *C6^−/−^* mice was not because of species incongruity between (human) Fc and (mouse) FcR; human IgG1 binds to all mouse FcγRs and can mediate Ab-mediated cellular cytotoxicity (ADCC) and Ab-dependent cellular phagocytosis (ADCP) with mouse effector cells in a manner similar to human cells (69). FH*/Fc with the complement-inactivating D270A/K322A Fc mutations was also ineffective in mice. Taken together with our prior observation of loss of FH*/Fc activity in *C1q^−/−^* mice (15), these data reiterate the role of classical pathway activation for FH*/Fc activity *in vivo*. A different *C6^−/−^* mouse constructed by back-crossing the naturally C6-deficient Peru-Coppock strain into the C3H/He background (70) and subsequently backcrossing the C3H/He *C6^−/−^* mice into the C57BL/6 background (71) showed impaired PMN function including defective phagocytosis and generation of reactive oxygen species (72). Whether the function of phagocytes in our *C6^−/−^* mice that were created by targeted deletion of *C6* directly in the C57BL/6 background is compromised remains to be determined. Nevertheless, collectively our data suggest that the classical and terminal pathways of complement were necessary for FH*/Fc function.

In summary, we have designed novel FH/Fc fusion proteins, expressed in tobacco plants, that show promising activity both *in vivo* and *in vitro* against *N. gonorrhoeae*. The modification of flexible linkers between FH* and Fc improves the potency of FH*/Fc. Intact classical and terminal complement pathways are required for FH*/Fc activity.

## Data Availability Statement

The raw data supporting the conclusions of this article will be made available by the authors, without undue reservation.

## Ethics Statement

The animal study was reviewed and approved by Institutional Animal Care and Use Committee at the University of Massachusetts Medical School.

## Author Contributions

JS, YT, KW, and SR designed the experiments, analyzed data and wrote the manuscript. JS, YT, BZ, SG, JM, and RBD performed the experiments and W-CS provided critical reagents. All authors contributed to the article and approved the submitted version.

## Funding

This work was supported by National Institutes of Health/National Institutes for Allergy and Infectious Disease grants R01 AI132296 and R44 AI147930 (both to SR and KW).

## Conflict of Interest

YT, KW, and JM are employed by the company Planet Biotechnology, Inc.

The remaining authors declare that the research was conducted in the absence of any commercial or financial relationships that could be construed as a potential conflict of interest.
