# Supplementary material for: Development of Complement Factor H–Based Immunotherapeutic Molecules in Tobacco Plants Against Multidrug-Resistant Neisseria gonorrhoeae
Source: Front Immunol. 2020 Oct 26;11:583305. doi: 10.3389/fimmu.2020.583305 (PMC7649208; doi:10.3389/fimmu.2020.583305)
Supplement: Supplementary file 1 [file DataSheet_1.pdf]

Supplemental Figure S1

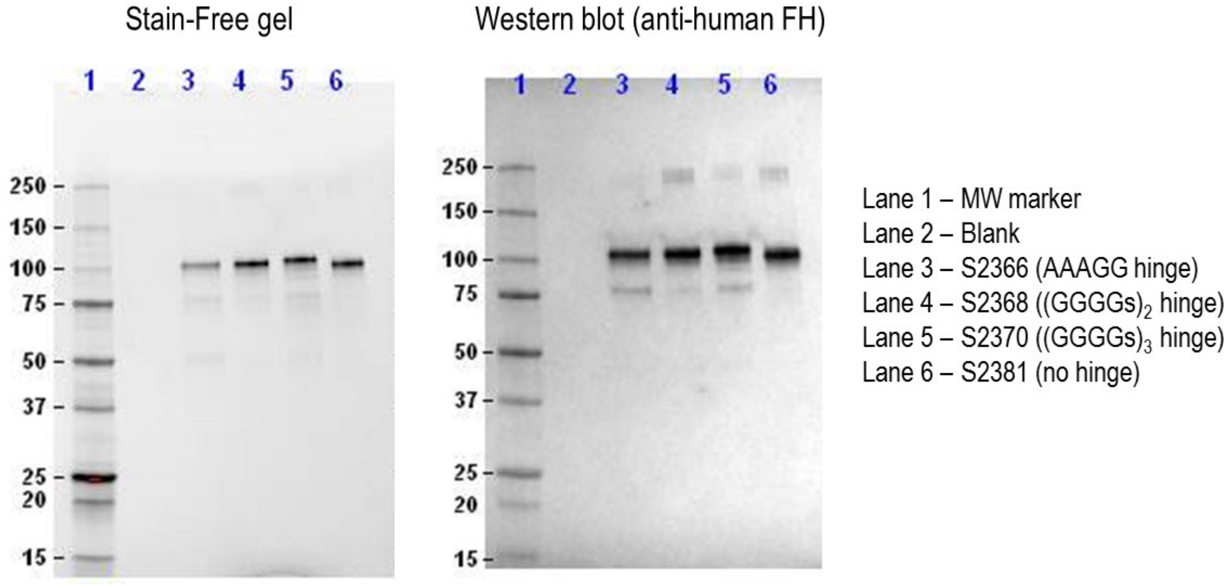

Supplemental Figure S1. Characterization of FH/Fc molecules. FH/Fc molecules S2366, S2368, S2370 and S2381 were run on SDS-PAGE gels and stained with Stain-Free (Bio Rad) (left figure) or transferred to a PVDF membrane by western blotting and probed with polyclonal goat anti-human factor H followed by donkey anti-goat IgG-Alkaline Phosphatase conjugate (right figure).
